# Cross-talk between airway and gut microbiome links to IgE responses to house dust mites in childhood airway allergies

**DOI:** 10.1038/s41598-020-70528-7

**Published:** 2020-08-10

**Authors:** Chih-Yung Chiu, Yi-Ling Chan, Ming-Han Tsai, Chia-Jung Wang, Meng-Han Chiang, Chun-Che Chiu, Shih-Chi Su

**Affiliations:** 1 Division of Pediatric Pulmonology, Department of Pediatrics, Chang Gung Memorial Hospital at Linkou, and Chang Gung University, Taoyuan, Taiwan; 2 Department of Emergency Medicine, Chang Gung Memorial Hospital at Linkou, Taoyuan, Taiwan; 3 Clinical Informatics and Medical Statistics Research Center, Chang Gung University, Taoyuan, Taiwan; 4 Department of Pediatrics, Chang Gung Memorial Hospital at Keelung, and Chang Gung University, Taoyuan, Taiwan; 5 Clinical Metabolomics Core Laboratory, Chang Gung Memorial Hospital at Linkou, College of Medicine, Chang Gung University, Taoyuan, Taiwan; 6 Whole-Genome Research Core Laboratory of Human Diseases, Chang Gung Memorial Hospital, Keelung, Taiwan; 7 Central Research Laboratory, XiaMen Chang Gung Hospital, Xiamen, China

**Keywords:** Asthma, Respiratory tract diseases, Paediatric research, Clinical microbiology, Microbiome

## Abstract

A connection between airway and gut microbiota related to allergen exposure in childhood allergies was not well addressed. We aimed to identify the microbiota alterations in the airway and gut related to mite-specific IgE responses in young children with airway allergies. This study enrolled 60 children, including 38 mite-sensitized children (20 rhinitis and 18 asthma), and 22 non-mite-sensitized healthy controls. Microbiome composition analysis of the throat swab and stool samples was performed using bacterial 16S rRNA sequencing. An integrative analysis of the airway and stool microbial profiling associated with IgE reactions in childhood allergic rhinitis and asthma was examined. The Chao1 and Shannon indices in the airway were significantly lower than those in the stool. Additionally, an inverse association of the airway microbial diversity with house dust mite (HDM) sensitization and allergic airway diseases was noted. Fecal IgE levels were positively correlated with the serum *Dermatophagoides pteronyssinus*- and *Dermatophagoides farinae*-specific IgE levels. Airway *Leptotrichia* spp. related to asthma were strongly correlated with fecal *Dorea* and *Ruminococcus* spp., which were inversely associated with fecal IgE levels and risk of allergic rhinitis. Moreover, four airway genera, *Campylobacter*, *Selenomonas*, *Tannerella*, and *Atopobium*, were negatively correlated with both serum mite-specific and fecal IgE levels. Among them, the airway *Selenomonas* and *Atopobium* spp. were positively correlated with stool *Blautia* and *Dorea* spp. related to asthma and allergic rhinitis, respectively. In conclusion, airway microbial dysbiosis in response to HDM and its cross-talk with the gut microbial community is related to allergic airway diseases in early childhood.

## Introduction

Dysbiosis in the gut microbiota is associated with several respiratory infections and lung diseases, including allergy and asthma^1,2^. There is accumulating evidence that a cross-talk exists between gut and lung. The upper gastrointestinal microbiota affects the development of the airway microbiota either by aspiration or metabolite production, leading to changes in the airway microenvironment^3^. Conversely, the dysbiosis in the lung microbiota is accompanied by that in the gut microbiota via bloodstream^4^. In a recent study, airway microbial dysbiosis appears to be strongly associated with sensitization to house dust mites (HDM), potentially contributing to allergic reactions and airway diseases^5^. However, its contribution to the gut-lung axis associated with allergies remains unclear.

HDM allergy with an elevated immunoglobulin E (IgE) level is strongly implicated in the allergic rhinitis and asthma pathogeneses in children^6^. Clinically, the comorbidity between these two conditions is high for perennial aeroallergens^7,8^. Furthermore, IgE is also produced locally in the gut and fecal IgE level could serve as a marker of allergic response to HDM^9^. The human microbiota mediates the mechanisms of the immune responses, which is essential for regulating mucosal inflammation^10^. The commensal organisms of the human microbiota that modulate the allergic response to HMD may differ in their molecular immune reactions, leading to different clinical manifestations and phenotypes. However, few studies have addressed the relationships of the microbiota and airway-gut connection in the clinical variants of allergic rhinitis and asthma phenotypes.

Microbial dysbiosis is increasingly being associated with the development and manifestations of allergic diseases^11^. A comprehensive understanding of the microbiome in response to allergens may provide insights into the pathological conditions affecting different phenotypes and a potential therapy for prevention or treatment of allergies. This study aimed to determine the airway and gut microbial profiles in children with airway allergies using 16S rRNA sequencing. The existence of the association between airway and gut microbiome and its implications in mediating HDM-specific IgE responses for childhood allergic rhinitis and asthma were also examined.

## Results

### Population characteristics

A total of sixty subjects were enrolled into this study, including 38 children with mite-sensitized allergic rhinitis (n = 20) and asthma (n = 18), and 22 healthy children. Table 1 presents the comparisons of baseline characteristics of children with rhinitis, asthma and healthy controls. Statistically significant differences were noted in the total serum and fecal, and *D. pteronyssinus*- and *D. farinae*-specific IgE levels among children with mite-sensitized rhinitis, asthma, and healthy controls (*P* < 0.05).

**Table 1:** Comparison of the clinical and epidemiologic characteristics between mite-sensitized rhinitis and asthma, and non-mite-sensitized healthy controls.

Characteristics	Mite-sensitized		*P*-value	Mite-sensitized	Non-mite-sensitized	*P*-value
	Rhinitis (n = 20)	Asthma (n = 18)		Total (n = 38)	Controls (n = 22)	
Age (yr)	4.35 ± 0.40	4.39 ± 0.53	0.848	4.37 ± 0.45	4.59 ± 0.36	0.178
Sex, male	14 (70.0%)	12 (66.7%)	0.460	26 (68.4%)	10 (45.5%)	0.266
Probiotic supplements	6 (30.0%)	10 (55.6%)	0.370	16 (42.1%)	8 (36.4%)	1.000
Cesarean delivery	6 (30.0%)	6 (33.3%)	1.000	12 (31.6%)	6 (27.3%)	1.000
Maternal atopy	6 (30.0%)	10 (55.6%)	0.524	16 (42.1%)	10 (45.5%)	0.858
Passive smoking	8 (40.0%)	10 (55.6%)	0.740	18 (47.4%)	12 (54.5%)	0.705
**Household income**						
Low, 500,000 NTD	10 (50.0%)	9 (50.0%)	0.276	19 (50.0%)	10 (45.5%)	0.152
Medium, 500,000–1,000,000 NTD	4 (20.0%)	7 (38.9%)		11 (28.9%)	12 (54.5%)	
High, > 1,000,000 NTD	6 (30.0%)	2 (11.1%)		8 (21.1%)	0 (0.0%)	
**Allergen-specific IgE, kU/L**						
*D. pteronyssinus*	54.58 ± 42.63	20.8 ± 34.07	0.065	38.58 ± 41.53	3.27 ± 8.69	**0.021**
*D. farinae*	38.63 ± 38.21	12.49 ± 18.64	0.095	26.25 ± 32.62	1.41 ± 3.33	**0.011**
Total serum IgE, kU/L	623.20 ± 632.22	164.52 ± 192.84	**0.043**	405.93 ± 521.29	44.92 ± 36.31	**0.008**
Total fecal IgE, kU/L	25.20 ± 32.45	4.63 ± 3.94	0.182	15.46 ± 25.39	1.92 ± 1.26	**0.003**

All *P*-values < 0.05, which is in bold, are significant.

Data shown are mean ± SD or number (%) of patients as appropriate.

*yr* year, *NTD* New Taiwan Dollar, *IgE* immunoglobulin E.

### Identification of the airway and stool bacterial community composition and abundance

**Figure 1:**
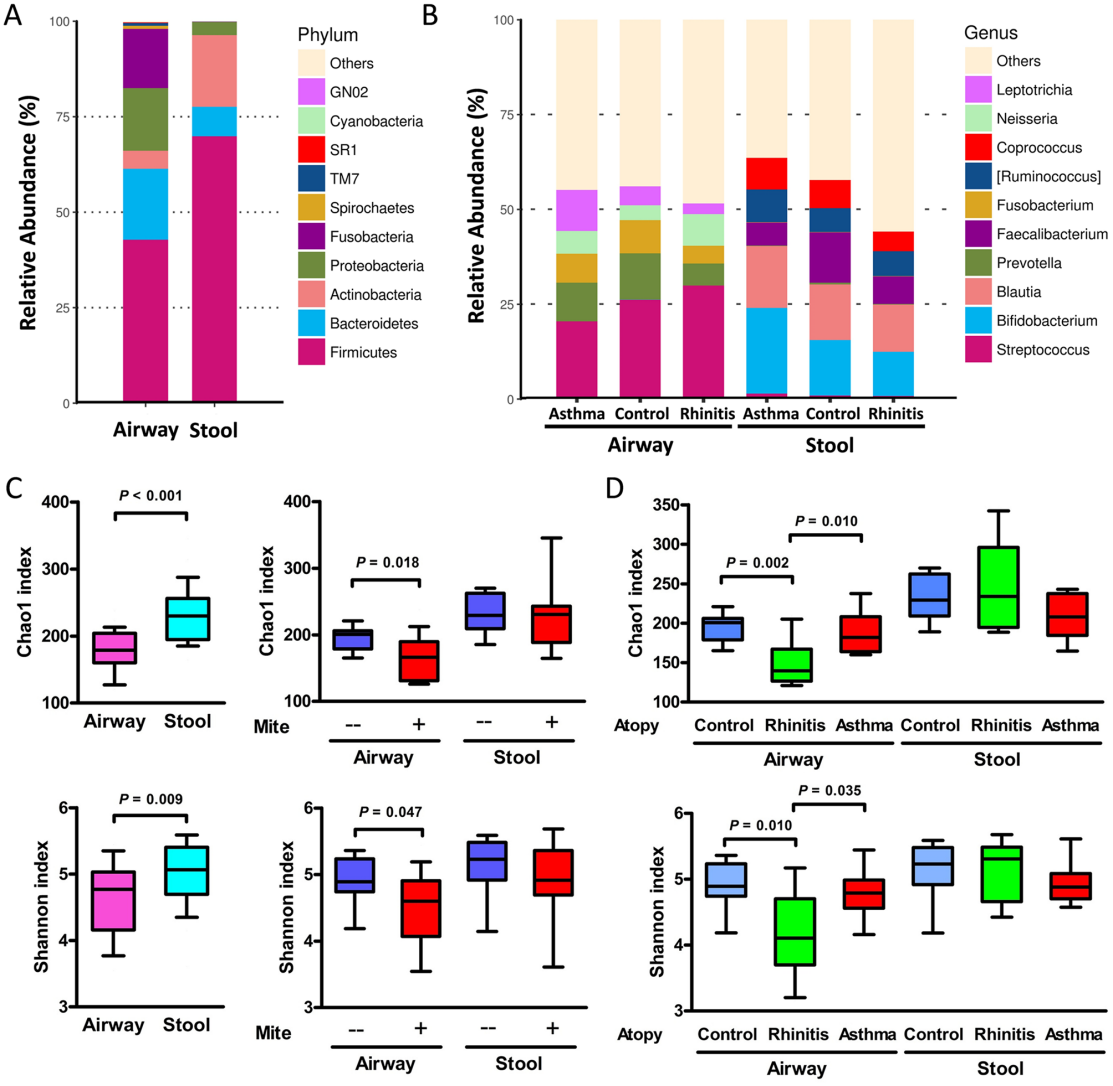
Airway and stool microbial composition and abundance at the phylum (**A**) and the genus level (**B**). Differences and comparisons of bacterial richness and diversity between airway and stool microbiota related to mite sensitization (**C**) and atopic diseases (**D**). Each bar represents the top ten enriched class categories ranked by the relative abundance in each group. Bacterial richness is calculated as the Chao1 index, and diversity is calculated as the Shannon index. The box-plot shows the median and the 10th, 25th, 75th and 90th percentile.

The taxonomic classification of the airway microbiota showed a high prevalence of phylum Firmicutes (42.8% of the total number of sequences obtained) followed by those of the phyla Bacteroidetes (18.6%), Proteobacteria (16.4%), and Fusobacteria (15.5%), whereas the phyla Firmicutes (69.9%), Actinobacteria (18.8%), Bacteroidetes (7.7%), and Proteobacteria (3.4%) were predominantly found in the stool microbiota (Fig. 1a). The most common genera in the airway microbiota were *Streptococcus* (24.5%), *Prevotella* (8.9%), and *Fusobacterium* (7.7%), whereas those in the stool microbiota were *Bifidobacterium* (16.0%), *Blautia* (14.3%), and *Faecalibacterium* (9.2%). The genera of airway and stool microbiota categorized by atopic diseases are shown in Fig. 1b.

### Bacterial richness and diversity categorized by atopic indices and diseases

Figure 1c shows the bacterial richness and diversity categorized by the sample types and atopic indices. Significantly lower Chao1 and Shannon indices were found in the airway microbiota than in the stool microbiota. In the airway microbiota, these indices were significantly reduced in children with mite sensitization, and were significantly lower in children with mite-sensitized rhinitis but not asthma than those in the healthy children without mite sensitization (Fig. 1d). However, in the stool microbiota, no difference was noted in the bacterial richness and diversity regarding the mite sensitization and its relevance to rhinitis and asthma. Furthermore, bacterial richness and diversity were not significantly different with regard to factors contributing to atopic diseases including sex, probiotics use, cesarean delivery, maternal atopy, passive smoking, and household income characteristics of the patients (Supplementary Figure S1).

### Abundance of bacterial taxa in the airway and stool for rhinitis and asthma

**Figure 2:**
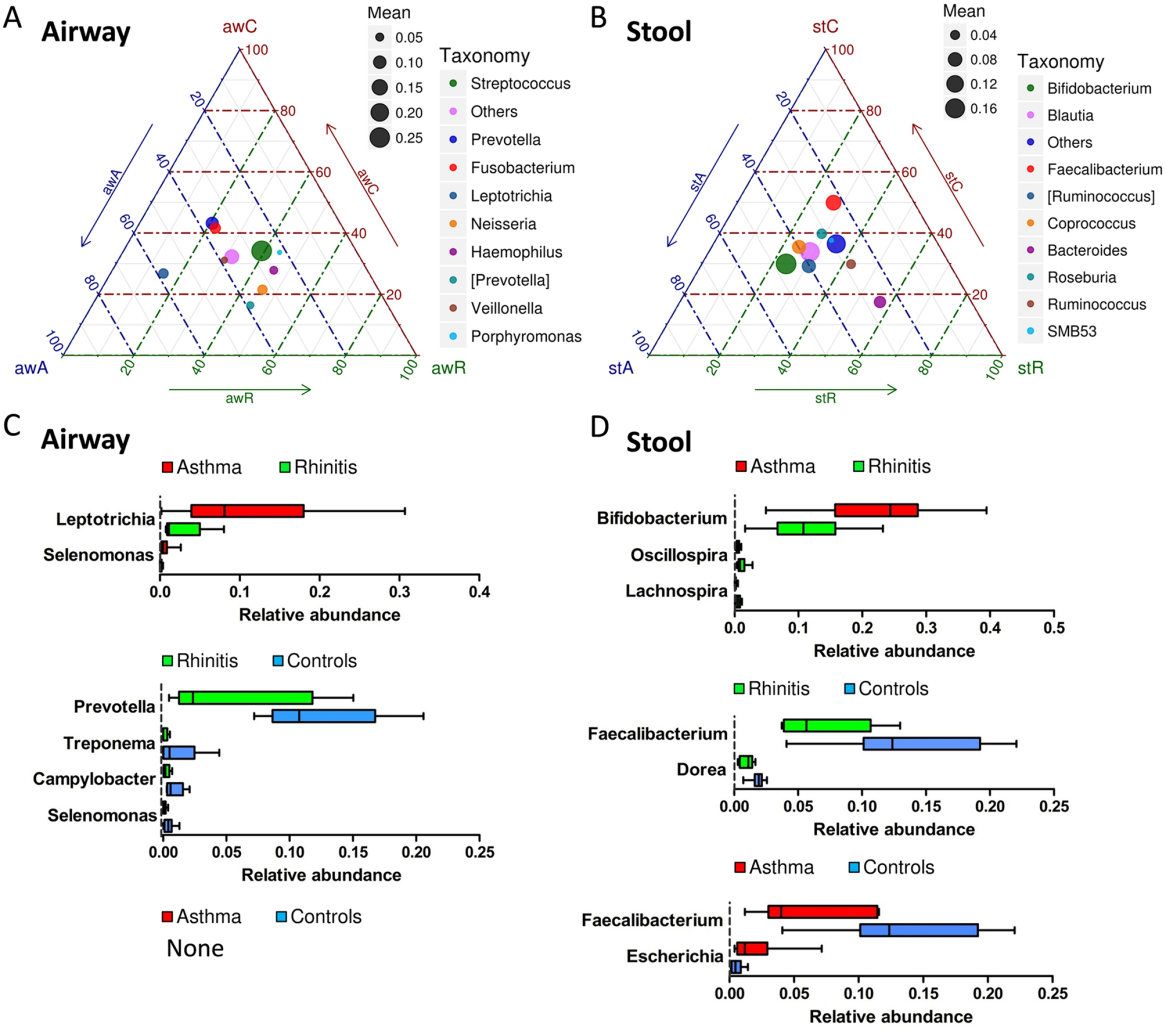
OTU distribution of ternary plots and significant differential expression of bacteria at the genus level across rhinitis, asthma, and healthy controls in airway (**A**, **C**) and stool (**B**, **D**). In ternary plots, the colors represent the genera to which corresponding OTUs were assigned. The size of plotted dots corresponds to the abundance of the OTUs with respect to each disease. The position of each circle is determined by the contribution of the indicated diseases to the total abundance. OTU, operational taxonomic unit.

The ternary plots of OTU distribution were generated to display the bacterial community composition and abundance contributing to rhinitis and asthma (Fig. 2a,b). The genera *Neisseria* and *Haemophilus* in the airway and *Bacteroides* in the stool microbiota were orientated toward mite-sensitized rhinitis. Contrarily, *Leptotrichia* in the airway and *Bifidobacterium*, *Ruminococcus*, and *Blautia* in the stool microbiota were orientated toward mite-sensitized asthma. Figure 2c,d show the differences in the abundance of the members of different genera among children with rhinitis and asthma and healthy controls using the MetaStat method. The genera *Prevotella*, *Treponema*, *Campylobacter*, and *Selenomonas* in the airway, and *Faecalibacterium* and *Dorea* in the stool microbiota were significantly reduced in children with mite-sensitized rhinitis compared with those in healthy controls. However, compared with children with mite-sensitized rhinitis, those with mite-sensitized asthma had significantly increased *Leptotrichia* and *Selenomonas* in the airway and *Bifidobacterium* in the stool microbiota.

### Association of airway and stool microbiota related to fecal and serum IgE levels

**Figure 3:**
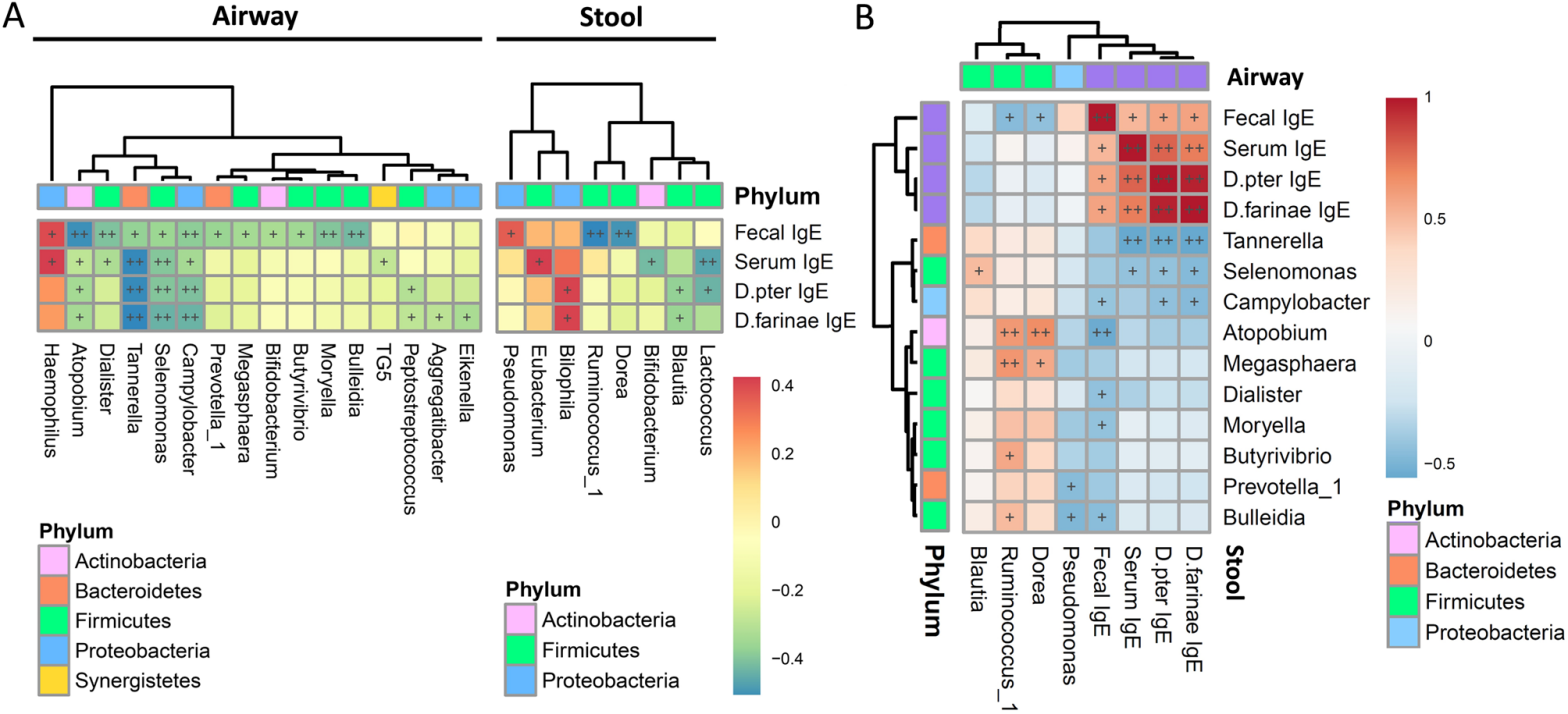
Heatmap of Spearman’s rank correlation coefficients between airway and stool microbiota associated with total fecal IgE and serum mite-specific IgE levels. Airway and stool genera of bacteria significantly correlated with fecal and serum IgE levels (**A**). FDR-adjusted correlations between IgE-related airway and stool genera of bacteria, and fecal and serum IgE levels (**B**). Color intensity represents the magnitude of correlation. Red color represents positive correlations; blue color represents negative correlations. + symbol means a *P*-value < 0.05; ++ symbol means a *P*-value < 0.01.

Spearman’s rank correlation coefficients between the airway and stool microbiota grouped by bacterial phylum are shown in Supplementary Figure S2. The genera *Atopobium*, *Megasphaera*, and *Leptotrichia* in the airway microbiota were significantly negatively correlated with *Akkermansia*, but positively correlated with *Dorea* and *Ruminococcus* in the stool microbiota. Figure 3a presents the correlations of the airway and stool microbiota with the fecal and serum IgE levels. Airway microbiota appeared to be mostly correlated with the total fecal IgE levels. *Atopobium*, *Bulleidia*, *Moryella*, and *Dialister* in the airway and *Dorea* and *Ruminococcus* in the stool microbiota were found to be negatively correlated with the total fecal IgE levels (*P* < 0.01). Conversely, *Haemophilus* in the airway and *Bilophila*, *Eubacterium*, and *Pseudomonas* in the stool microbiota were positively correlated with the IgE levels. Furthermore, *Tannerella*, *Selenomonas*, and *Campylobacter* in the airway microbiota were significantly negatively correlated with the total fecal, serum and mite-specific IgE levels.

Figure 3b depicts the FDR-adjusted correlations between the IgE-related airway and stool genera of bacteria. Their detailed scatter plots of these correlations are shown in Supplementary Figure S3. The total fecal IgE levels were significantly positively correlated with those of specific IgE levels to *D. pteronyssinus* and *D. farinae* (*P* < 0.01). Additionally, *Selenomonas* in the airway was positively correlated with *Blautia* in the stool microbiota. Furthermore, *Atopobium* and *Megasphaera* in the airway were significantly positively correlated with both *Dorea* and *Ruminococcus*.

## Discussion

Gut microbiota dysbiosis involved in lung diseases in the gut-lung axis has been widely observed, but the contribution of the allergen exposure on the airway microbiota to this axis remains unclear. This study has demonstrated a modulation of HDM exposure on the microbiota composition alteration predominantly in the airway. An association of the particular subsets of airway microbial dysbiosis and gut microbial community related to allergies supports the existence of cross-talk between the gut and lung contributing to allergic airway diseases.

The human microbiota is extremely diverse and differs significantly at several body sites^12^. The gut is the most densely colonized surface, whereas airway is one of the least-populated surfaces of the human body. In this study, similar to the gut, the most predominant phylum detected in the airways was Firmicutes. However, the diversity of the airway microbiota was significantly lower than that of the gut microbiota. In addition, an inverse association between the HDM sensitization and microbial diversity of the airway but not of the gut was noted, indicating the alteration of the airway microbiota composition may specifically represent the impact of aeroallergen exposure on allergies.

Allergic reactions to HDM play a significant role in various childhood atopic diseases^13^. In this study, the fecal IgE levels were strongly correlated with the serum total and mite-specific IgE levels, indicating the gut-lung connection and the possibility of a relation of allergic diseases between gut and lung^1^. Dysbiosis in gut microbiota has been implicated in several lung diseases, including allergy and asthma^14^. However, mite-sensitized airway diseases appeared to be only associated with microbial dysbiosis in the airway than in the gut^5,9^. Despite the fact that these findings support the existence of a cross-talk between the gut and lung, the changes in the airway microbial community related to HDM may take part in the composition of the gut microbiota in childhood rhinitis and asthma.

A modulation between microbial dysbiosis and responses to allergens potentially contributes to the susceptibility to allergic airway diseases^5,9^. *Leptotrichia* spp. in the airway was found to be significantly in the interaction of mite sensitization with asthma but not with rhinitis^5^, which is consistent with the findings of this study. Nevertheless, in the gut, a reduction of *Dorea* and *Ruminococcus* spp. inversely associated with the fecal IgE levels was strongly related to children with mite-sensitized rhinitis but not asthma in this study. However, airway *Leptotrichia* spp. was strongly correlated with gut *Dorea* and *Ruminococcus* spp., indicating that the microbial dysbiosis linked to HDM between gut and lung may influence the phenotypes observed in allergic airway diseases.

The microbial dysbiosis and interactions with the gut-lung axis and its implications have a complex effect on health and airway diseases^3^. In this study, airway *Campylobacter*, *Selenomonas*, *Tannerella*, and *Atopobium* were strongly correlated with both serum mite-specific and fecal IgE levels. Among them, *Campylobacter* and *Selenomonas* were significantly reduced in children with rhinitis. Most importantly, airway *Selenomonas* and *Atopobium* spp. were positively related to stool *Blautia* and *Dorea* spp., respectively. In the intestinal tract, *Blautia* spp. primarily provides beneficial anti-inflammatory effects^15^, whereas *Dorea* spp. has shown to be associated with childhood rhinitis^9^. These results suggest that the particular subsets of airway microbial dysbiosis associated HDM-specific IgE responses can interact with corresponding gut microbiome, contributing to the susceptibility to allergic airway diseases.

Gut microbiota coordinates to shape mucosal immunity and contribute to modulating inflammation and allergic reactions^16^. In this study, reduced *Faecalibacterium* spp. accompanied with increased *Escherichia* spp. in the gut microbiota were significantly associated with a higher risk of asthma. The genus *Faecalibacterium*, a butyrate-producing bacterium, plays an essential role in maintaining the intestinal epithelium integrity for anti-inflammatory activities^17,18^. Furthermore, butyrate can acidify the local gastrointestinal microenvironment, making it less suitable for the overgrowth of pathogenic species such as *Escherichia*^19^. However, both the genera *Faecalibacterium* and *Escherichia* were not correlated with IgE levels in this study. These observations elucidate a potential role of certain gut bacteria in a reaction involving other components of the immune system apart from IgE antibodies for childhood asthma.

Fecal microbiota composition is dynamic and individualized depending on the influence of diet, exposition to ingested probiotic bacteria, and intestinal environmental conditions. Bifidobacteria in the probiotics are particularly effective at modulating the immune response and protecting against allergic diseases^20^. However, the probiotic bacteria could be mostly retrieved in the subjects’ stools without affecting microbial diversity^21^. In this study, the genus *Bifidobacterium* spp. was conversely enriched in asthma children, which could be particularly explained by the high usage rate of yogurt and probiotic supplements for allergy as in this study.

One major limitation of this study is it`s relatively small sample size that may not be representative of the entire population. The cross-sectional design of our study also limits the ability to make causal inferences. However, the analysis of outcomes for an age-matched comparison design eliminates the dissimilarities in the microbial compositions across a wide range of age groups. Any contamination will also not have systematically influenced our results because of using the same protocols and procedures for cases and control subjects. Most importantly, the oropharynx is constantly exposed to microbes from both the upper and lower respiratory tract, where would be representative of the microbes related to airway diseases. Moreover, an integrated airway and gut microbiome analysis in this study provides a comprehensive overview of the regulatory network of microbiome in allergic airway diseases.

In conclusion, an inverse association of the airway microbial diversity with HDM sensitization and allergic airway diseases suggests that the modulation between airway microbiota composition and HDM responses may potentially play a role in the susceptibility to airway allergies. A significant association between the particular subsets of HDM-associated airway microbial dysbiosis and gut microbial community corroborates the cross-talk between the gut and lung contributing to allergic airway diseases. However, further research is required for functional studies to elucidate the molecular mechanisms of these associations.

## Methods

### Study population

A cross-sectional case–control study nested within PATCH birth cohort recruited since 2007 was conducted to investigate the gut and airway microbial profiles in children aged 4–5 years diagnosed with mite-sensitized asthma alone or rhinitis alone for the first time, and healthy controls. Atopic diseases were evaluated using the ISAAC questionnaire and were physician-diagnosed by the same pediatric pulmonologist at the outpatient clinics^5,22^. Healthy children without a history of atopic conditions or infections were enrolled as controls. Children suffered an upper airway infection or chronic viral infection mimicking atopic diseases were excluded. Information regarding demographic data and factors related to atopic diseases were collected. This study was approved by the Ethics Committee of Chang Gung Memory Hospital (No. 104-3757B). All experiments and methods in this study were performed in accordance with relevant guidelines and regulations, and written informed consent was obtained from the parents or guardians of all study subjects.

### Collections of throat swab and stool samples

Samples of throat swab and stool were collected in subjects without receiving antibiotics therapy for at least 4 weeks^23^. Sterile cotton swabs were rubbed by physician around the oropharynx with swab rotation at least three times to collect throat swab samples^5^. Throat swab were frozen and stored at − 80 °C immediately after sampling. Clean specimen bottles were used to collect fresh stools by parents from each child according to the instructions on proper method of collection^9^. The parents were telephone-informed to collect stool samples 3 days before an outpatient appointment. The stool samples were stored in the freezer at home directly after collection and carefully transported to our laboratory in cooled condition using ice cubes, and were then stored at − 80 °C until use.

### Measurement of total and allergen-specific IgE levels

Total serum and allergen-specific serum IgE levels were examined as described in our previous study^24^. Total serum IgE levels were measured by ImmunoCAP (Phadia, Uppsala, Sweden), and specific IgE levels to aeroallergens (*Dermatophagoides pteronyssinus* and *Dermatophagoides farinae*) were determined using a commercial assay for IgE (ImmunoCAP Phadiatop Infant; Phadia). Mite sensitization was defined as *D. pteronyssinus*- or *D. farinae*-specific IgE levels  17.5 kU/L (class 4–6) to confirm the presence of sensitization^25^. Total fecal levels of IgE were measured using Immunoglobulin E ELISA Kit (Immundiagnostik AG, Bensheim, Germany) according to the manufacturer’s instructions.

### 16S rRNA gene amplification and sequencing

Throat swab bacterial DNA was extracted using a FastDNA Spin Kit for Soil (MP Biomedical, Solon, OH, USA) and fecal bacterial DNA was extracted from the same amount of feces (0.5 g) using a FastDNA Spin Kit for Feces (MP Biomedical, Solon, OH, USA) following the manufacturer’s instructions. The variable region V3-V4 encoded for 16S rRNA gene was amplified by using bacteria/archaeal primer 341F/805R for throat swab samples and 341F/806R for stool samples respectively with the barcodes^5,26^. Sequencing libraries were generated using the NEBNext Ultra II DNA Library Prep Kit for Illumina (New England BioLabs, USA) and subjected to sequencing on an Illumina HiSeq 2,500 platform (Illumina, Inc., San Diego, CA, USA) according to the manufacturer’s instructions. DNA extraction kits without clinical sample as negative controls and reagent controls for potential kit contamination were performed, but did not detect any amplicons in both controls for subsequent 16S rNRA sequencing. The sequence raw data and mapping file for all the samples included in this study have been originally deposited in Figshare (https://figshare.com/s/e337e0b26af010885553).

### Microbiome data analysis

Analysis of microbiome data was performed using the software ‘‘Quantitative Insights into Microbial Ecology’’ (QIIME 1.9.1)^27^. Assembled sequences were clustered into operational taxonomic units (OTUs) using Uparse software at 97% sequence identity, and taxonomy classification was assigned based on the Greengenes 16S rRNA Database version 13.8^28^. To normalize variation in sequence depth across samples, OTUs abundance information was rarefied to the minimum sequence depth using the QIIME script (single_rarefaction.py). As described in our previous study^5^, richness of each sample was calculated with the Chao1 index and diversity accounting for both relative abundance and evenness was evaluated with Shannon index. The ternary plot of OTU relative abundance was generated to visualize the distribution of bacterial communities among rhinitis, asthma, and healthy controls with the ggtern extension package to R software (Lucent Technologies, NJ, USA, version 3.5.1)^29^. This plot showed the proportion of the abundance of the genera per group as positions in the triangle using barycentric coordinates. The Metastat method was used to test the differences in the microbial composition of microbiome between groups with adjusted *P*-values for multiple comparisons^30^. In the species with significant differences, OTUs presented in at least 10% of samples with a mean proportional abundance of > 0.01% were further selected^31^.

### Statistical analysis

Comparisons of baseline characteristics between children with rhinitis and asthma, and between with mite-sensitized allergic airway diseases and healthy controls were done with non-parametric tests such as Mann–Whitney U test, chi-square test, and Fisher’s exact test. The correlation coefficients between the gut and airway bacterial compositions, and fecal and serum specific IgE levels were calculated using Spearman’s correlation test with false discovery rate (FDR) adjustment for multiple measurements in R software (Lucent Technologies, NJ, USA, version 3.3.1). Statistical analysis was performed by using the Statistical Package for the Social Sciences (SPSS Statistics for Windows Version 20.0; Armonk, NY). All statistical hypothesis tests were 2-tailed and a *P*-value < 0.05 was considered significant.

## Supplementary information

Supplementary file1

## Data Availability

The datasets generated during and/or analyzed during the current study are not publicly available duo to the personal privacy of subjects but are available from the corresponding author on reasonable request.
