# Supplementary material for: Cross-talk between airway and gut microbiome links to IgE responses to house dust mites in childhood airway allergies
Source: Sci Rep. 2020 Aug 10;10:13449. doi: 10.1038/s41598-020-70528-7 (PMC7417544; doi:10.1038/s41598-020-70528-7)
Supplement: Supplementary file 1 — Supplementary file1 [file 41598_2020_70528_MOESM1_ESM.docx]

**Supplementary information**

**Cross-talk between airway and gut microbiome links to IgE responses to house dust mites in childhood airway allergies**

Chih-Yung Chiu, Yi-Ling Chan, Ming-Han Tsai, Chia-Jung Wang, Meng-Han Chiang, Chun-Che Chiu, and Shih-Chi Su

**
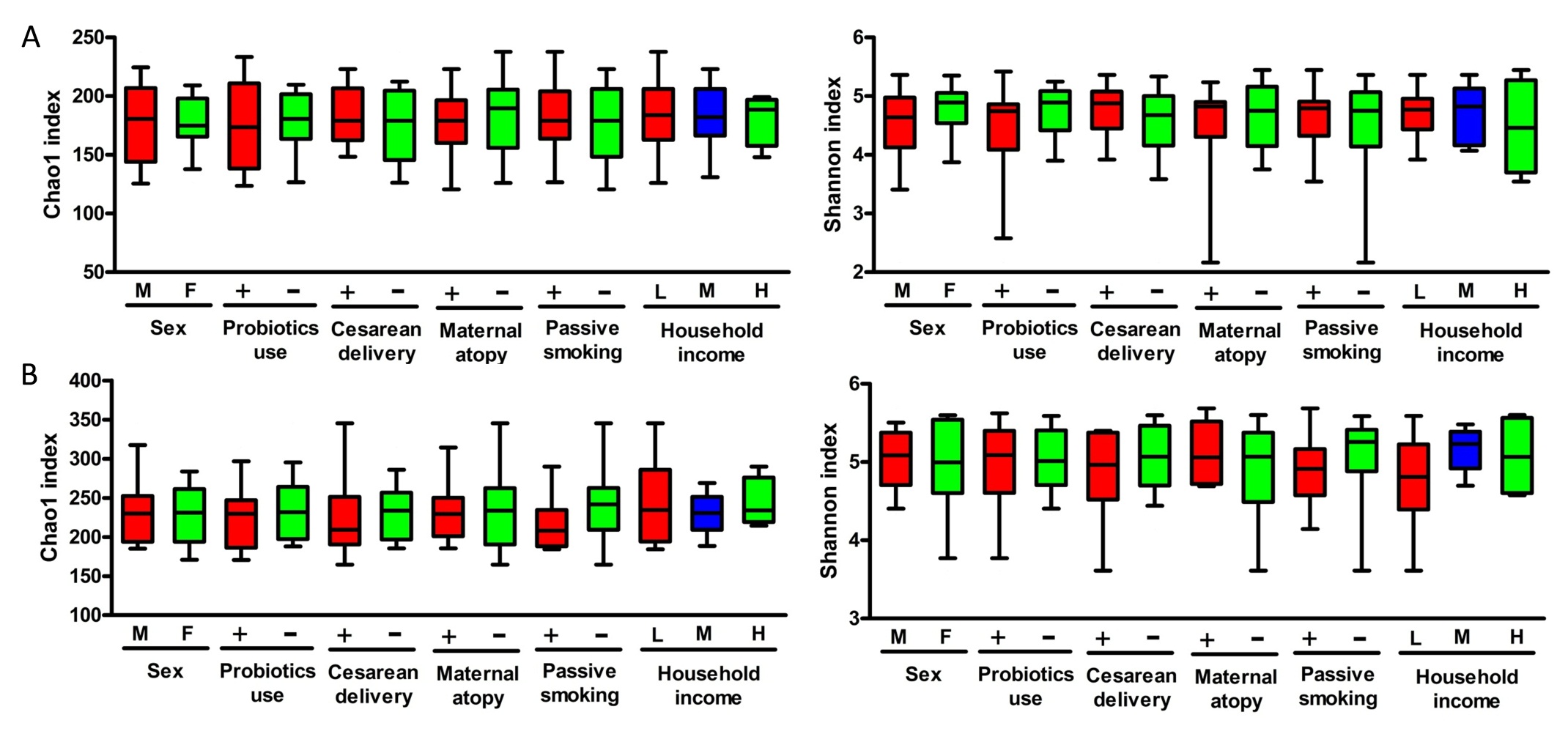
**

**Supplementary Figure S1.** Differences and comparisons of species richness (Chao1 index) and diversity (Shannon index) for factors including sex, probiotics use, cesarean delivery, maternal atopy, passive smoking, and household income in the airway (A) and the stool (B).


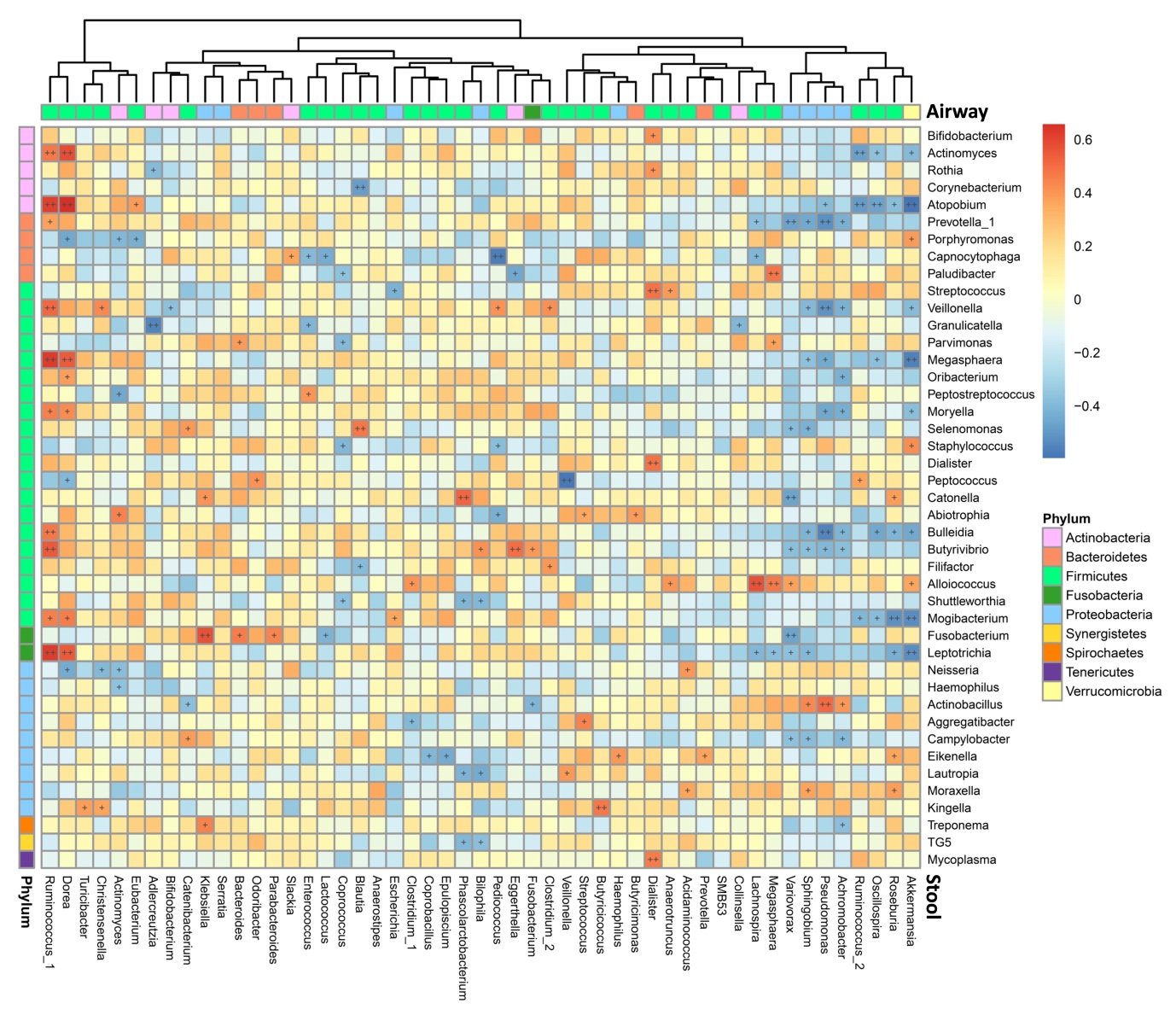


**Supplementary Figure S2.** Heatmap of Spearman’s rank correlation coefficients between airway and stool genera of bacteria. Only OTUs present in at least 10% of samples with a mean proportional abundance of 0.01%. Color intensity represents the magnitude of correlation. Red color represents positive correlations; green color represents negative correlations. + symbol means a *P*-value < 0.05; ++ symbol means a *P*-value < 0.01. OTU, operational taxonomic unit.


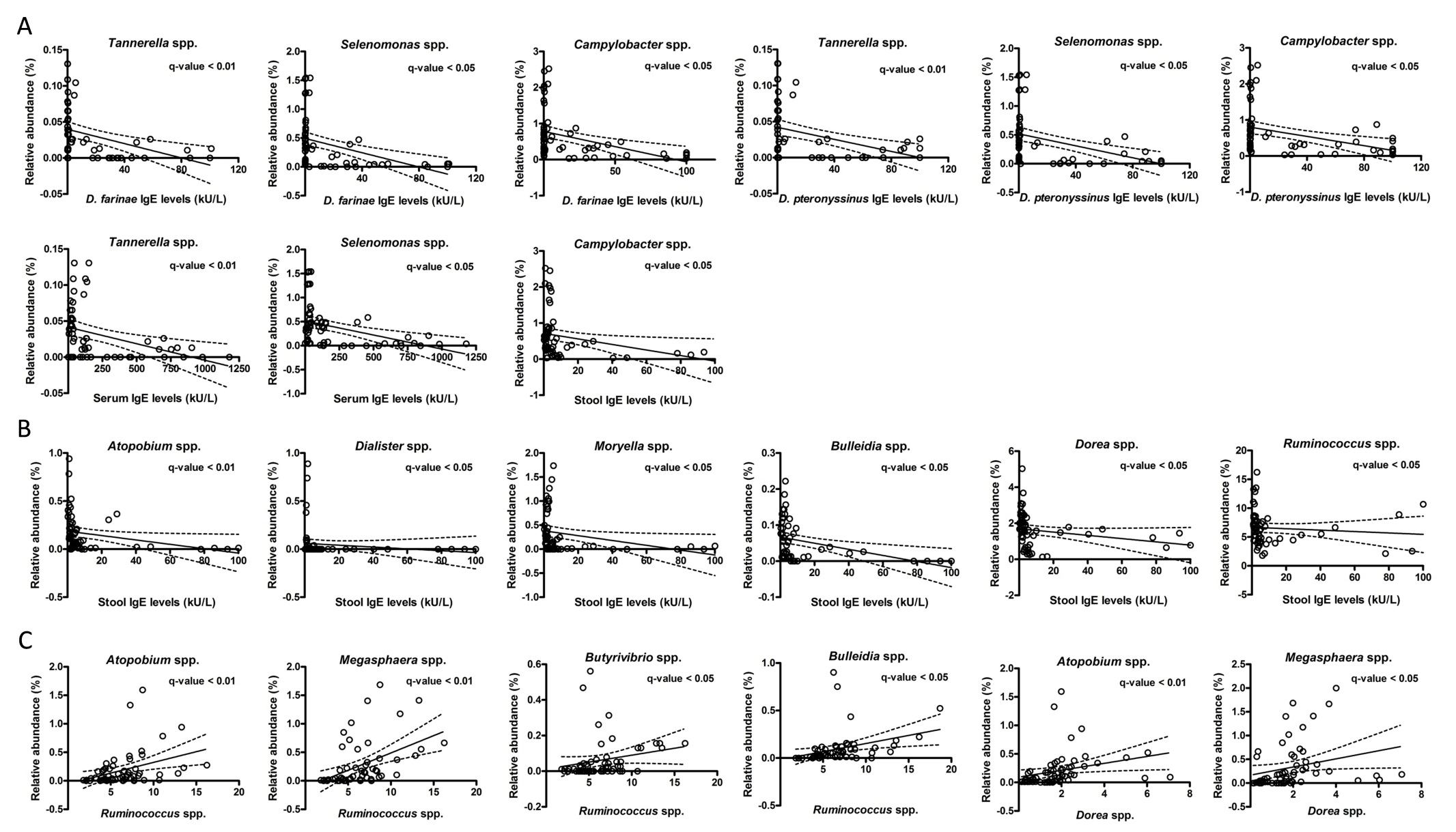


**Supplementary Figure S3.** Scatter plots of correlations between the genera *Tannerella*, *Selenomonas*, and *Campylobacter* and allergen-specific IgE levels (A), and between the genera *Atopobium*, *Dialister*, *Moryella*, *Bulleidia*, *Dorea*, and *Ruminococcus* and stool IgE levels (B), and between the genera in the airway and *Ruminococcus* and *Dorea* spp. in the stool (C).
